# Structures of type IV pilins from *Thermus thermophilus* demonstrate similarities with type II secretion system pseudopilins

**DOI:** 10.1016/j.jsb.2016.08.006

**Published:** 2016-12

**Authors:** Vijaykumar Karuppiah, Angela Thistlethwaite, Jeremy P. Derrick

**Affiliations:** Michael Smith Building, Faculty of Life Sciences, University of Manchester, Oxford Road, Manchester, UK

**Keywords:** Pilus, Type II secretion system, X-ray crystallography, Thermophile, Natural competence

## Abstract

Type IV pilins are proteins which form polymers that extend from the surface of the bacterial cell; they are involved in mediating a wide variety of functions, including adhesion, motility and natural competence. Here we describe the determination of the crystal structures of three type IVa pilins proteins from the thermophile *Thermus thermophilus*. They form part of a cluster of pilus-like proteins within the genome; our results show that one, Tt1222, is very closely related to the main structural type IV pilin, PilA4. The other two, Tt1218 and Tt1219, also adopt canonical pilin-like folds but, interestingly, are most closely related to the structures of the type II secretion system pseudopilins, EpsI/GspI and XcpW/GspJ. GspI and GspJ have been shown to form a complex with another pseudopilin, GspK, and this heterotrimeric complex is known to play a key role in initiating assembly of a pseudopilus which is thought to drive the secretion process. The structural similarity of Tt1218 and Tt1219 to GspI and GspJ suggests that they might work in a similar way, to deliver functions associated with type IV pili in *T. thermophilus*, such as natural competence.

## 1 Introduction

Type IV pili (T4P)^1^ are long, surface-exposed filaments which extend from the surfaces of bacterial cells. They are found extensively in Gram-negative bacteria, as well as Gram-positive organisms and archaea (Berry and Pelicic, 2015, Giltner et al., 2012). T4P are responsible for mediating a wide range of different functions, including adhesion to host cells, uptake of DNA in natural competence and twitching motility (Berry and Pelicic, 2015, Giltner et al., 2012). The fibres consist of multiple copies of pilin proteins, which are non-covalently associated and are added from the base. T4P are capable of retraction, a process which involves rapid disassembly of the fibre and deposition of the pilin subunits into the inner membrane. T4P pilus retraction is associated with twitching motility, a phenomenon which supports migration of bacteria across solid surfaces (Burrows, 2005). T4P are composed of both major and minor pilin components, although major pilins comprise the majority of the pilin subunits within each fibre. However, minor pilins, which are present in much lower quantities, are also known to play a critical role in T4P function; for example, genetic knock-outs of the minor pilins *pilHIJK* in *N. meningitidis* produce a non-piliated phenotype, although evidence suggested that they are not critical for pilus assembly (Carbonnelle et al., 2006). Another minor pilin, PilX in *N. meningitidis*, controls T4P-mediated attachment and signalling to endothelial cells (Brissac et al., 2012, Helaine et al., 2007, Helaine et al., 2005). PilV in *Neisseria gonorrhoeae* is essential for adherence, but not other pilus-mediated functions (Winther-Larsen et al., 2001). Recent evidence also suggests that some minor pilins in *Neisseria* could exert their influence through modulation of the surface numbers of T4P (Imhaus and Dumenil, 2014). Parallel studies in *Pseudomonas aeruginosa* have provided evidence that minor pilins initiate pilus assembly, by formation of a complex, and promote display of PilY1, a non-pilin protein which mediates T4P functions (Nguyen et al., 2015).

Current models for T4P assembly have the individual pilin subunits located in the inner membrane, before assembly into the pilus fibre (Berry and Pelicic, 2015). Crystal structures of several T4P pilin subunits have established that they adopt a canonical fold, consisting of an N-terminal α-helix, around 50 residues in length, packed against a β-sheet (Craig and Li, 2008). This arrangement forms a ‘ladle-like’ structure, with the N-terminal helix acting as the handle. Models for fibre assembly, from electron microscopy data, have the N-terminal helices associated non-covalently in the core of the fibre and related by an axial displacement of 8–10 Å (Giltner et al., 2012). The N-terminal helix, outside of the globular domain, is hydrophobic, which is thought to promote solubility of the pilin within the inner membrane. This portion of the pilin is generally removed for structural studies, to improve solubility.

It is well established that mutations in T4P biogenesis proteins frequently lead to a non-competent phenotype in bacteria which are capable of natural transformation (Allemand et al., 2012, Burton and Dubnau, 2010, Seitz and Blokesch, 2013). The precise nature of the connection between T4P formation and the uptake of DNA during natural transformation is unclear, however. Current models for DNA uptake in both Gram positive and Gram negative bacteria involve the formation of a pilus or pseudopilus which functions, in concert with specialised inner membrane proteins, to promote transfer of DNA into the cell (Burton and Dubnau, 2010, Seitz and Blokesch, 2013). Cehovin *et al.* reported the identification of a specialised minor pilin in *Neisseria meningitidis*, ComP, which has a type IV pilin-like fold and is capable of binding to DNA in a sequence-specific manner, suggesting that some other minor type IV pilins could also function in DNA recognition (Cehovin et al., 2013).

**Fig. 1:**
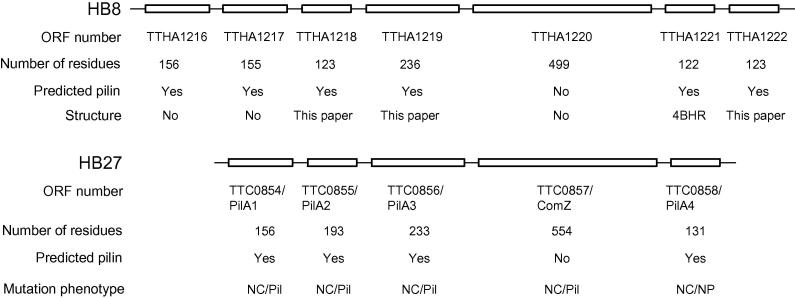
Type IV pilin clusters in *T. thermophilus* HB8 and HB27. Number of residues indicated are before predicted processing by PilFind (Imam et al., 2011). ORF names for the HB27 pilins are those given by Friedrich et al. (Friedrich et al., 2003). For the mutation phenotypes, NC is non-competent, Pil is piliated and NP is non-piliated.

*Thermus thermophilus* strains HB27 and HB8, along with other *Thermus spp*., are capable of type IV pilus formation and natural transformation (Averhoff, 2009). Orthologs of the main T4P biogenesis proteins are present, including the ATPase PilF (Collins et al., 2013), the integral inner membrane proteins PilO and PilN, and the outer membrane protein PilQ (Averhoff, 2009). A recent study by cryoelectron microscopy has provided low resolution structural details of the *T. thermophilus* T4P assembly machinery in open and closed states from whole cell tomography (Gold et al., 2015). A locus of 4 pilin-like genes in *T. thermophilus* strain HB27 was identified by Freidrich et al., which they designated PilA1-4 (Friedrich et al., 2003) (Fig. 1). Individual deletion of PilA1, 2 and 3 gave non-competent phenotypes, although piliation was unaffected, suggesting involvement in DNA uptake, rather than pilus assembly. Mutation of PilA4 resulted in a non-competent, non-piliated phenotype, suggesting that it is the major structural subunit pilin in the pilus fibre. We have conducted several structural studies of several T4P biogenesis proteins from *T. thermophilus* HB8, which we have found tractable to crystallization (Karuppiah and Derrick, 2011, Karuppiah et al., 2010, Karuppiah et al., 2013). This has included the structure of the type IV pilin TTHA1221 which, we showed, bound specifically to the detergent solubilised PilMNO inner membrane complex (Karuppiah et al., 2013). TTHA1221 forms part of a series of predicted pilin-like proteins which are found in part of the *T. thermophilus* HB8 genome (Fig. 1). Sequence diversity among pilins is considerable- the sequence identity of the pilins from HB8 and HB27 shown in Fig. 1 is around 20%, with the exception of TTHA1217/TTC0854, at 37%. This emphasizes the importance of structural studies, in order to elicit possible functional roles for individual pilins, and their possible functions in natural competence and T4P assembly.

## 2 Materials and methods

### 2.1 Pilin expression and purification

Synthetic expression constructs for the *T. thermophilus* HB8 pilins were designed with omission of the residues predicted to lie within the hydrophobic portion of the N-terminal helix, so that expression started at residue 36 for Tt1218^36-123^, 33 for Tt1219^33-236^ and 38 for Tt1222^38-123^ (residues are numbered starting from the methionine in the predicted sequence of the pilin pre-protein). Constructs were optimized for codon usage in *E. coli*, synthesized (GeneArt) and subcloned into pET22b vector using the restriction sites NcoI and XhoI. The final expressed peptide encoded the PelB leader sequence at the N-terminus, followed by the truncated pilin and hexahistidine tag at the C-terminus. The following protocol was used for all three pilins. Each expression plasmid was transformed into T7 express cells (New England Biolabs) and inoculated into 50 ml of terrific broth (TB) media containing 100 μg/ml of ampicillin and grown at 37 °C for 3 h. The starter culture was diluted into 2 L of TB media and grown at 37 °C until the OD_600_ reached 0.6–0.8. At this point, the flasks were cooled and IPTG was added to a final concentration of 0.1 mM and incubated at 16 °C for 15 h. Cells were harvested and resuspended in Buffer A (25 mM Tris-HCl pH 8, 100 mM NaCl) supplemented with 1X protease inhibitor cocktail (Roche), DNase (10 μg/ml) and RNase (5 μg/ml). The cells were lysed using a probe sonicator, for 10 min, with the probe set at 40% amplitude. The lysate was centrifuged at 30,000*g* for 30 min and the supernatant was passed through a 0.2 μm filter. 4 mL of Ni-NTA resin (Qiagen) was added to the filtered supernatant and incubated at 4 °C for 1 h with gentle rolling. The resin was packed onto an empty gravity-flow column and washed with Buffer A plus 40 mM imidazole. The pilin was eluted by application of Buffer A plus 200 mM imidazole. Eluted fractions were concentrated (Vivaspin, 5 kDa cutoff) and injected onto a Superdex 75 10/300 column (GE Healthcare) pre-equilibrated with Buffer A. The fractions from the chromatogram peaks corresponding to the predicted monomer elution volumes were pooled and concentrated to 7–10 mg/ml. For expression with selenomethionine enrichment, the plasmid encoding Tt1218^36-123^ was transformed into B834 (DE3) (Novagen) and cells grown in modified M9 minimal media (Molecular Dimensions). The purification procedure was identical to that described for the native protein, with the exception that 1 mM DTT was included in Buffer A throughout.

### 2.2 Crystallization and data collection

Crystallization trials were carried out in MRC 2-well plates using a Mosquito robot (TTP Labtech), with a 1:1 drop ratio and incubated at 20 °C. Eight commercially available crystallization screens (Molecular Dimensions and Microlytic) were used for initial trials. Tt1218^36-123^ native crystals were grown by mixing 400 nl of protein (7 mg/ml) with 400 nl of well solution, which comprised 0.1 M Hepes pH 7, 1 M succinic acid and 1% PEG (w/v) 2000 MME. Selenomethionine Tt1218^36-123^ crystals were grown by the same method, except that the well solution contained 0.2 M LiCl, 0.1 M Hepes pH 7 and 20% (w/v) PEG 6000. Crystals were cryoprotected by washing for 5 min in well solution supplemented with 20% glycerol (v/v), before flash cooling in liquid nitrogen. Tt1218^36-123^ crystals, both native and selenomethionine, initially diffracted X-rays to a resolution of 4–6 Å, but annealing the crystals for 2–4 s at the beamlines improved the resolution considerably. Tt1219^33-236^ crystals were grown by mixing 400 nl of protein (8 mg/ml) with 400 nl 0.01 M zinc chloride, 0.1 M sodium acetate pH 5 and 20% (w/v) PEG 6000. A native dataset to 1.85 Å resolution was collected, in a *P*2_1_ spacegroup crystal form. A second crystal form was identified, in spacegroup *C*2, which diffracted to a lower resolution (2.3 Å). The crystals were cryoprotected in a similar manner to the procedure described for Tt1218^36-123^. For phasing of Tt1219^33-236^, crystals were incubated for 10 min in the well solution supplemented with freshly prepared 0.4 M KI, which was also included in the cryoprotectant. Tt1222^38-123^ crystals were grown by mixing 200 nl of protein solution (7–10 mg/ml) with 200 nl of well solution, which comprised 0.1 M sodium acetate pH 4.6 and 2 M ammonium sulfate. Repeated attempts to derivatize Tt1222^38-123^ crystals with heavy metals using platinum, mercury and gold salts were unsuccessful; fortunately, incubation of the crystals with 0.4 M KI, in a similar manner to the procedure for Tt1219^33-236^, yielded an iodide derivative which permitted effective phasing. Diffraction data were collected at Diamond Light Source beamlines I02, I04 and I24. Datasets were processed by automated pipeline implemented in xia2 (Winter et al., 2013), using XDS (Kabsch, 2010), and relevant data collection statistics are summarised in Table 1, Table 2, Table 3.

### 2.3 Structure determination

In the case of Tt1219^33-236^, experimental phases were derived from data from four different crystals, which were merged and scaled using Aimless (Evans and Murshudov, 2013), as implemented in the CCP4 suite (Winn et al., 2011). For all three pilin structures, automated heavy atom substructure identification was combined with experimental phasing and model building using Autosol (Terwilliger et al., 2009), as implemented in PHENIX (Adams et al., 2010). For Tt1219^33-236^, phase calculation was assisted with autoSHARP (Vonrhein et al., 2007). In each case the partially completed pilin structures were used as search models for molecular replacement, against their respective higher resolution native datasets, using Phaser (McCoy et al., 2007). Automated model building by Autobuild (Terwilliger et al., 2008) built majority of the residues. The structures were completed by manual model building in Coot (Emsley et al., 2010) and refined using phenix.refine (Afonine et al., 2012) and refmac (Murshudov et al., 2011). The structures were validated using Molprobity (Chen et al., 2010) and PDB_REDO (Joosten et al., 2014). The relevant phasing statistics and refinement parameters are detailed in Table 1, Table 2, Table 3. Coordinates and structure factors have been deposited in the Protein Data Bank, with the following accession codes: 5G2F (Tt1222^38-123^), 5G25 (Tt1218^36-123^), 5G23 (Tt1219^33-236^, *P*2_1_ form), 5G24 (Tt1219^33-236^, *C*2 form).

## 3 Results

### 3.1 Cluster of pilin-like proteins within the T. thermophilus HB8 genome

We identified a cluster of type IV pilin-like genes within the *T. thermophilus* HB8 genome, using the program PilFind (Imam et al., 2011). Type IV pilins are highly diverse in sequence, with the exception of the N-terminal helix, which is hydrophobic and contains a highly conserved Glu or Asp residue at position 5 (Craig and Li, 2008). PilFind identified 6 out of the 7 ORFs as type IV pilins, the exception being TTHA1220, which also has a predicted α-helix at its N-terminus but is considerably larger (Fig. 1). For expression and purification we adopted the common practice of deleting the hydrophobic portion of the N-terminal helix and ensured correct folding by including a PelB leader sequence, to direct the expressed protein into the *E. coli* periplasm. We conducted a systematic investigation by expressing all 7 proteins individually, followed by purification and crystallization trials. The structure of TTHA1221 has been reported previously (Karuppiah et al., 2013). Three of the remaining six, TTHA1218, TTHA1219 and TTHA1222, yielded crystals of truncated forms which enabled us to solve their structures (designated here as Tt1218^36-123^, Tt1219^33-236^ and Tt1222^38-123^). Of the remaining three, TTHA1216 expressed poorly, TTHA1217 failed to crystallize and TTHA1220 yielded thin plate crystals which diffracted to low resolution.

### 3.2 Structure of Tt1222^38-123^

**Fig. 2:**
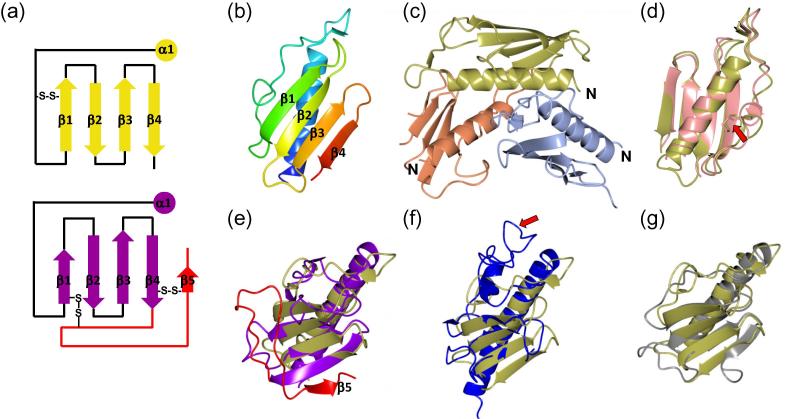
Structures of Tt1222^38-123^ and structural homologs (a) topology fold diagram for Tt1222^38-123^ (gold) and ComP from *Neisseria meningitidis* (PDB 5HZ7; purple residues, 373–467; red residues (DD loop), 468–490). (b) Tt1222^38-123^ monomer, with a colour gradient from blue (N terminus) to red (C terminus), with β-strands numbered. (c) Tt1222^38-123^ asymmetric unit trimer, with N-termini indicated. Chain A is light blue, B gold and C orange (d) Chain B from Tt1222^38-123^ (gold) superimposed onto chain A from TTHA1221 (PDB 4BHR) in pink. The red arrow shows the conserved disulphide bonds. (e) Superposition of ComP T4P domain (PDB 5HZ7, coloured as in a) on to Tt1222^38-123^ (gold). (f) Superposition of GspG (PDB 3G20, blue) on to Tt1222^38-123^ (gold). The red arrow indicates the longer loop between α1 and β1. (g) Superposition of PilBac1(PDB 4D40, grey) on to Tt1222^38-123^ (gold). Superpositions and figures were generated using CCP4MG (Potterton et al., 2004). (For interpretation of the references to colour in this figure legend, the reader is referred to the web version of this article.)

Tt1222^38-123^ adopts a typical pilin-like fold, with a central α-helix packed against a 4-stranded antiparallel β-sheet (Fig.2a & b). There is a trimer in the asymmetric unit, with two Tt1222^38-123^ molecules (chains A and B) aligned approximately parallel and a third (chain C) bound in the opposite orientation (Fig.2c). Analysis of crystallographic packing by PISA (Krissinel and Henrick, 2007) highlighted the interaction between chains A and B as significantly larger than other interfaces, with a buried surface area of 600 Å^2^, 10 inter-chain hydrogen bonds, 4 salt bridges and a Complexation Significance Score (CSS) score of 0.4. Chain A is transformed onto B by a rotation of 138° and translation of 16.5 Å, possibly indicative of an interaction preserved in fibre assembly. For comparison, a right handed 1-start helical model for *Neisseria gonorrhoeae* T4P has a 10.5 Å rise and 100.8° azimuthal rotation (Craig et al., 2006). However, such an inference should be treated with caution, given that we observed Tt1222^38-123^ to be monomeric by size exclusion chromatography (SEC; not shown).

A search for homologous structures was conducted using HHPred (Soding et al., 2005) and DALI (Holm and Rosenstrom, 2010); the results of the closest structural homologs are summarized in Table 4. Unsurprisingly, the most similar structure in the PDB was another *T. thermophilus* T4Pa pilin, PilA4 (designated as TTHA1221 in Fig. 1). Previously we noted that the structure of TTHA1221, which was the first pilin structure from *Thermus* to be reported, had two major points of difference from other T4Pa pilins (Karuppiah et al., 2013). First, the D region- a C-terminal segment which is commonly found in T4Pa pilins- is absent. Second, other T4P pilins have a disulphide bond in the D region: it is missing in TTHA1221, and replaced by one between the α/β loop and the first β-strand (Karuppiah et al., 2013). Both these features were preserved in Tt1222^38-123^: the two pilin structures superimposed with an r.m.s.d. of 1.24 Å over 77 matched residues (Fig.2d), suggesting that they may have a common evolutionary ancestor, and perhaps either could be incorporated as the main pilin into the assembled T4P fibre in *T. thermophilus* HB8.

Given the potential involvement of Tt1222^38-123^ in natural competence, it is interesting that a closely related structure, as judged by r.m.s.d. after superposition, was ComP from *N. meningitidis*. The ComP fold is more extensive than Tt1222^38-123^, with an additional β-strand (β5) and a loop which wraps around the back of the β-sheet (shown in red in Fig2e). Berry *et al*. carried out NMR titrations of ComP with specific DNA uptake sequences (DUS), in order to identify which regions of the pilin are involved in recognition of nucleic acid (Berry et al., 2016). Chemical shift changes were identified in the DD region (the long loop from β5), and the tip of the loop between β1 and β2. Tt1222^38-123^ therefore appears to lack a specific structural feature, the DD region, necessary for recognition of DNA. Furthermore, calculation of electrostatic surfaces for Tt1222^38-123^ did not reveal a distribution of positive charge which would be characteristic of nucleic acid binding, as noted for ComP (Berry et al., 2016).

Pseudopilins play a critical role in type II secretion, and are thought to operate through a piston-type mechanism to convey transported substrate across the periplasm and outer membrane. Pseudopilin structures are similar to type IV pilins: the GspG T2SS pseudopilin from *E. coli* has a similar fold to Tt1222^38-123^, although the β-sheet is less extensive, it lacks the fourth β-strand and it forms a more elaborate loop between α1 and β1 (Fig2f).

The similarity between TTHA1221 and the Pil_Bac1_ pilin from *Shewanella oneidensis* has been noted previously by Gorgel et al. (2015) and it is, at present, the type IV pilin from another organism with the most structural similarity to TTHA1221 and Tt1222^38-123^ (Fig2g). Gorgel *et al*. also remarked on the absence of a kink in the α-helix, generally caused by a helix-breaking residue (eg., Pro). The equivalent residue in this position in Tt1222^38-123^ is Asn49 and, in common with Pil_Bac1_, there is no bend to the helix at this position. We carried out a similar pilus modeling procedure to that described by Gorgel et al., which built a fibre based on the *N. gonorrhoeae* pilus (PDB 2HIL; (Craig et al., 2006)). The modeling indicated that the structure of Tt1222^38-123^ could be incorporated into the pilus fibre model, although some repacking of the core helices would be required to satisfy steric constraints (not shown).

### 3.3 Structure of Tt1218^36-123^

**Fig. 3:**
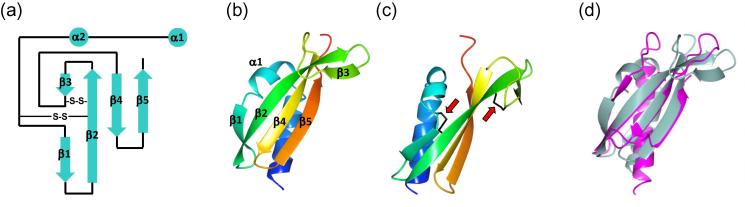
Structure of Tt1218^36-123^ and comparison with EspI (a) Topology fold diagram for Tt1218^36-123^. (b) Tt1218^36-123^ monomer, with a colour gradient from N-terminus (blue) to C-terminus (red). (c) An second view of Tt1218^36-123^, rotated by approximately 90° from (b), showing the location of the two disulphide bonds, indicated by red arrows (d) Tt1218^36-123^ (grey) superimposed on EpsI from PDB 3CFI (in magenta). Figures and superpositions were carried out using CCP4MG (Potterton et al., 2004). (For interpretation of the references to colour in this figure legend, the reader is referred to the web version of this article.)

Although the structure of Tt1218^36-123^ identifies it as recognizably part of the pilin family, it exhibits a different fold topology from Tt1222^38-123^ (Fig.3a and b). The first β-strand is formed from the loop between the α-helix and what would be β1 in Tt1222^38-123^. Formation of this strand may be assisted by a disulphide bond which links the loop to β2 in Tt1218^36-123^ (which is β1 in Tt1222^38-123^; left arrow in Fig.3c). This location is similar to the conserved disulphide observed in TTHA1221 and Tt1222^38-123^ (Fig.2d), hinting that this may be a common structural feature of *Thermus* pilins. The structure also contains a second disulphide bond, which links β2 and β3 in the equivalent location to the D loop (right arrow in Fig.3c). Both Tt1218^36-123^ and Tt1222^38-123^ form 4-stranded β-sheets, but the location of the strands is displaced between the two: a short α-helix and β1 in Tt1218^36-123^ are formed in place of the loop between the α-helix and β1 in Tt1222^38-123^; β2 in Tt1218^36-123^ aligns with β1 in Tt1222^38-123^, and there is no equivalent of β4 from Tt1222^38-123^ in Tt1218^36-123^.

We used the protein structure comparison service PDBeFold at the European Bioinformatics Institute (Krissinel and Henrick, 2004), HHPred (Soding et al., 2005) and DALI (Holm and Rosenstrom, 2010) to search for pilins with similar folds (Table 4). The closest structure identified was the type II secretion pseudopilin EpsI (PDB 3CFI) from *Vibrio vulnificus*, which superimposed on Tt1218^36-123^ with an r.m.s.d. of 2.1 Å over 59 residues (Fig.3d; Table 4). The structure of EpsI from *Vibrio vulnificus* was originally determined by Yanez et al. in complex with another pseudopilin, EpsJ: they form a heterodimer in solution, as well as in the crystalline state (Yanez et al., 2008). Subsequently, the *V. vulnificus* EpsI/J complex was also crystallized in complex with a nanobody (Lam et al., 2009); the relative orientation of EpsI with respect to EpsJ differs between the two structures, although the general helix-helix interaction between the two pseudopilins is preserved. The structures of EspI and EpsJ are similar to GspI and GspJ noted in the heterotrimeric GspIJK complex from *E. coli*, published earlier (Korotkov and Hol, 2008). These observations prompted us to examine the potential interaction between Tt1218^36-123^ and Tt1219^33-236^; none was observed by SEC, however (not shown).

### 3.4 Structure of Tt1219^33-236^

**Fig. 4:**
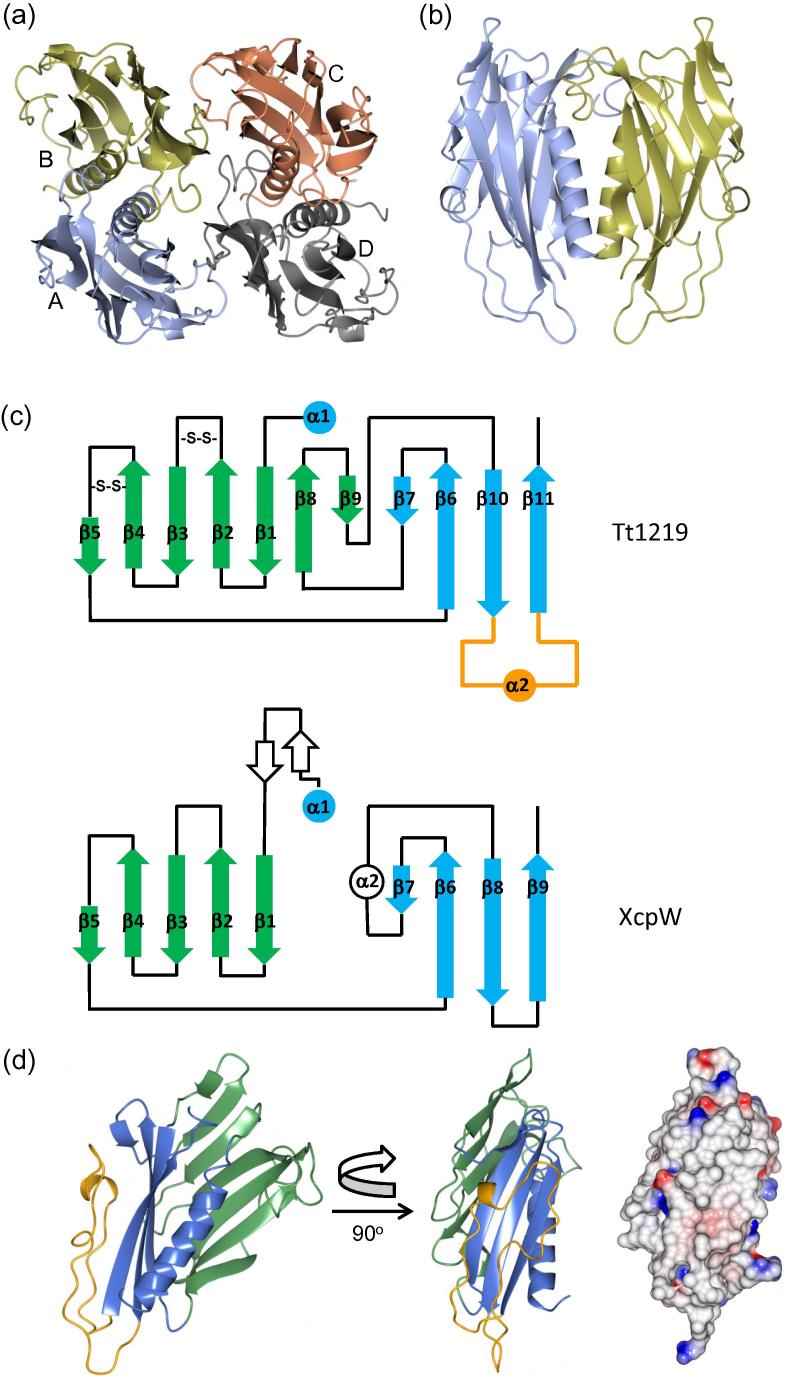
Structure of Tt1219^33-236^. (a) Tt1219^33-236^ asymmetric unit tetramer from *P*2_1_ native crystal form; individual chains are labeled. (b) Tt1219^33-236^ dimer, showing the interface between chains A and B (coloured as (a)). (c) Topology diagrams of Tt1219^33-236^ and XcpW. Blue is the conserved β-sheet; green is the variable β-sheet and the loop region between residues 184 and 221 is shown in orange. (d) Two orthogonal views of Tt1219^33-236^ monomer, coloured as in (c). The far right panel shows an electrostatic charge surface, of the Tt1219^33-236^ monomer, in the same orientation as the middle panel, calculated using CCP4MG (Potterton et al., 2004). (For interpretation of the references to colour in this figure legend, the reader is referred to the web version of this article.)

Tt1219^33-236^ crystallized in two different crystal forms; the higher resolution form, at 1.85 Å resolution in spacegroup *P*2_1_, has four molecules in the asymmetric unit, arranged as a dimer of dimers (Fig.4a). The interfaces between monomers were analyzed by PISA (Krissinel and Henrick, 2007), which revealed that the interface areas between chains A and B, and its equivalent between C and D, were substantial, with a mean of 1488 Å^2^ (the next largest interface area was 439 Å^2^). The CSS score for both interfaces was 1.0, suggesting that Tt1219^33-236^ forms a dimer in solution. The monomer-monomer interface is dominated by the helix-helix interaction across the non-crystallographic 2-fold symmetry axis (Fig.4b). The second crystal form, in spacegroup C2, has a dimer in the asymmetric unit; crucially, the interface between the A and B chain dimer is preserved in this crystal form, providing further evidence that this dimer is not a crystallization artifact. Formation of a dimer in solution by Tt1219^33-236^ was investigated by SEC, which showed elution peaks for both monomer and dimer, as well as a third peak in the void volume, which appeared to be associated with contaminants in the preparation (Supplementary Fig. 1).

Interrogation of PDBeFold (Krissinel and Henrick, 2004) with the structure of Tt1219^33-236^ gave a top ‘hit’ of a type II secretion system pseudopilin, XcpW, from *Pseudomonas aeruginosa* (r.m.s.d. 2.0 Å over 111 residues; Table 4). XcpW is closely related in structure to two other type II secretion pseudopilins, EpsJ from *Vibrio vulnificus* (Yanez et al., 2008), and GspJ from enterotoxigenic *Escherichia coli* (Korotkov and Hol, 2008). This group of pseudopilins consists of the canonical ‘core’ type IV pilin fold of an α-helix packed against a 4-stranded β-sheet, referred to here as the ‘conserved’ sheet, consistent with the terminology used by Franz et al. (Franz et al., 2011) (shown in blue in Fig.4c & d). In addition, a second sheet is formed, termed the ‘variable’, which wraps around the helix from the side (green in Fig.4c & d). Tt1219^33-236^ forms a more complex fold topology than XcpW and the related T2SS pseudopilins, with two main differences. First, two additional β-strands, β8 and β9, are inserted into the variable β-sheet, which extend this structural feature into the same spatial location occupied by the short α2 helix in XcpW (Fig.4c). Second, Tt1219^33-236^ contains a long loop, from residue 184 in β10 to 221 in β11, which folds back over the external surface of the conserved sheet (marked in orange in Figs4c & d). This has the effect of covering the external face of the conserved sheet. The hydrophobic properties of this face of the conserved β-sheet have been remarked on previously for XcpW, with the suggestion that it could serve as the site for packing of another pseudopilin (Franz et al., 2011). Interestingly, addition of this loop in Tt1219^33-236^ preserves this feature of a hydrophobic surface, as apparent from the calculated electrostatic surface (see far right of Fig.4d). Tt1219^33-236^ is also stabilized by two disulphide bonds, between β2/β3, and β4/β5; both lie within the variable β-sheet. Type IV pilins incorporate disulphide bonds, presumably to confer additional stability, although they are absent from T2SS pseudopilins, which preferentially use Ca^2+^ to perform a similar function (Korotkov et al., 2009).

## 4 Discussion

Although minor pilins are present in low abundance, they exert substantial influences on T4P assembly and function (Giltner et al., 2012). Minor pilins can be divided into two groups, ‘core’ and ‘non-core’; genes for the former are typically found in polycistronic operons within the genome and they align with the equivalent type II secretion system (T2SS) pseudopilins (Giltner et al., 2012). Non-core minor pilins have a variety of functions and typically modulate T4P formation, rather than being primarily involved in pilus fibre assembly, through control of surface pilus density, for example (Imhaus and Dumenil, 2014). The three pilins whose structures we report in this paper were derived from *T. thermophilus* strain HB8: we know little of their function(s), including whether they would be minor core or non-core. Parallel work on *T. thermophilus* HB27 has shown that the pilin-like proteins PilA1, 2 and 3 from this strain can be mutated with loss of natural competence, but not piliation (Friedrich et al., 2003). These would therefore be identified as ‘non-core’; it is possible therefore that TTHA1218, 1219 and 1222 from HB8 fall into the same category. If so, they could be involved in formation of a ‘competence pilus’ (Seitz and Blokesch, 2013) and thus play a role in DNA uptake by natural transformation (Averhoff, 2009). We found no obvious structural features in Tt1222^38-123^, Tt1218^36-123^ or Tt1219^33-236^ which were suggestive of a classical DNA binding motif. However, this also appears to be the case with *N. meningitidis* ComP, where a model for the complex suggests an unorthodox direct interaction of two loop regions within the grooves of the double helix (Berry et al., 2016). The primary receptor for binding of DNA during natural transformation in *Thermus* could be a small monomeric protein, ComEA (Averhoff, 2009). Indeed, the crystal structure of ComEA shows a classical helix-turn-helix motif (PDB 2DUY), consistent with this hypothesis. That observation does not exclude the possibility that minor pilins are also involved in DNA recognition, however, although their relationship with ComEA and the transmembrane protein ComEC, which is also conserved in other naturally transformable bacteria, is unclear.

Disulfide bonds are particularly common in proteins from thermophiles; it has been suggested that this is one mechanism by which proteins can be stabilized at high temperatures (Beeby et al., 2005). This might explain why the minor pilins studied here tend to have more disulphide bridges than their counterparts from mesophilic bacteria. Interestingly, the role of calcium ion binding in stabilization of T2SS pseudopilins has also been noted (Korotkov et al., 2009). Aside from these features, there was a remarkably high degree of similarity in structure between the type IV pilins described here and their T2SS counterparts (Table 4), suggesting that they form a highly adaptable platform which can be modified to different secretion tasks.

Type IV pilins share a common structural fold with pseudopilins from the T2SS, one of several similarities between the two secretion systems, possibly indicative of a common evolutionary origin. Our results show that TTHA1218 and TTHA1219 are closer in structure to T2SS pseudopilins than to other type IV pilins for which structures are currently available. This provides further reinforcement for the hypothesis that the basic mechanisms underpinning fibre assembly are similar for T4P biogenesis and type II secretion. Indeed, it has been shown that the T4P *E. coli* minor pilins PpdAB-YgdB-PpdC can prime assembly of the T2SS *Klebsiella* PulG pseudopilin which was lost in a *pulHIJK* mutant (Cisneros et al., 2012a). The type IV core minor pilin subunits are proposed to correspond to the four minor pseudopilins involved in T2S (PulI, J, K and H). Recent work has produced evidence to indicate that binding of PulI to PulIJ initiates pseudopilus assembly, followed by binding of PulK which results in partial displacement of the complex from the inner membrane (Cisneros et al., 2012b). Current models for the mechanism of the T2SS invoke the formation of a pseudopilus, which operates as part of a piston-like mechanism (McLaughlin et al., 2012). Assembly of the pseudopilus is driven by ATP hydrolysis; it is thought to interact with the secreted substrate and power opening of the entrance to the outer membrane secretin, followed by transit across the outer membrane.

In *P. aeruginosa*, the type IV core minor pilins, FimU, PilV, PilW and PilX, are clustered in an operon; determination of the crystal structure of FimU revealed close similarity to the T2SS pseudopilin GspH, despite the low sequence homology between the two proteins (Nguyen et al., 2015). This work complements our observations reported here, in that we have identified two type IV pilins from *Thermus* which are most closely related at a structural level to two other T2SS pseudopilins, GspI and GspJ. Both sets of observations therefore point to a remarkable conservation of minor pilin structure, so that some T4P minor pilins are more closely related to their equivalent T2SS pseudopilin counterparts than they are to other type IV pilins.

**Fig. 5:**
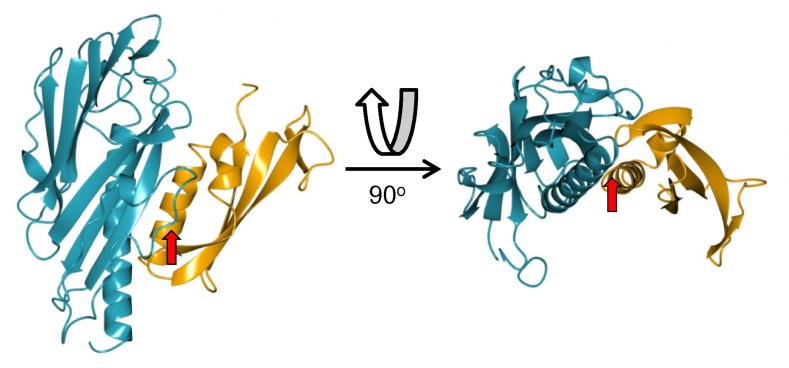
Model of Tt1218^36-123^ and Tt1219^33-236^ heterodimeric complex. Tt1219^33-236^ (light blue) and Tt121836-123 (orange) were superimposed on the coordinates of GspI and GspJ respectively (PDB 3CI0). Close contacts are indicated with red arrows for the β10 and β11 loop (left hand image) and the N-terminus of the α-helix of Tt1218^36-123^ (right hand image). Figures and superpositions were carried out using CCP4MG (Potterton et al., 2004). (For interpretation of the references to colour in this figure legend, the reader is referred to the web version of this article.)

The crystal structure of the GspI/GspJ/GspK heterotrimeric complex from *E. coli* revealed an arrowhead-like structure (Korotkov and Hol, 2008); the α-helices from each subunit interact in a similar fashion to that found in models for assembled T4P (Giltner et al., 2012). To test whether Tt1218^36-123^ and Tt1219^33-236^ could plausibly form a heterodimer, we modelled both structures on to their GspI/J counterparts in the GspIJK structure (Fig 5). Inspection of the structure suggested that its formation is plausible but would require two structural changes to alleviate minor steric clashes. The first is the tip of the loop between β10 and β11; the second is the N-terminus of the α-helix in Tt1218^36-123^. Both changes could be relatively easily accommodated by minor adjustments. GspK is the largest of the subunits in the GspIJK heterotrimer, and was proposed to form the tip of the pseudopilus, as further incorporation of pilin subunits into the fibre would be sterically blocked (Korotkov and Hol, 2008). We therefore examined TTHA1220, which lies adjacent to TTHA1218 and TTHA1219 in the HB8 genome, as a possible GspK ortholog (Fig. 1). TTHA1220 is more than twice the size of the preceding two pilins, but we think it is unlikely to adopt a similar fold to GspK or, indeed, the type IV pilins PilX or PilK. There is no significant sequence homology between TTHA1220 and any of these proteins. Secondary structure predictions, and attempts at homology modeling with the GspK structure as a template, did not produce convincing results. Moreover, analysis of the TTHA1220 sequence by PilFind (Imam et al., 2011) failed to identify it as a pilin at all (the predicted transmembrane segment lies too far from the predicted prepilin peptidase cleavage position). It is possible that a GspK ortholog exists elsewhere in the *T. thermophilus* genome, or that TTHA1220 substitutes for GspK in some way, but this is unclear at present.

Recent advances in whole cell tomography have produced novel insights into the assembly of the T4P biogenesis system *in vivo* (Chang et al., 2016, Gold et al., 2015). The work by Chang et al., in particular, places minor pilins at the inception of the nascent pilus fibre, which is built from the bottom (Chang et al., 2016). This is in agreement with the proposal by Imhaus and Dumenil, that minor pilins are not incorporated into the assembled fibre, but play a role in assembly (Imhaus and Dumenil, 2014) (although this view is controversial (Nguyen et al., 2015)). An attraction of this hypothesis, however, is that it provides an explanation for the similarity in minor pilin and pseudopilin structures- that they may provide similar functions in initiating and regulating T4P assembly. Further work will be needed to establish exactly what those functions are, and how they relate to the sequence of T4P assembly.

## Figures and Tables

**Table 1:** Crystal structure of Tt1218^36-123^: data collection and refinement statistics.

	Native	Selenomethione
*Data collection*		
Space group	*F*4_1_32	*P*4_2_32
Unit cell parameters	a = 156.6 Å	a = 78.4 Å
X-ray source and wavelength (Å)	DLS^a^ IO2 (0.90)	DLS IO4 (0.97)
Number of crystals	1	1
Resolution range (Å)	55.36–2.30 (2.36–2.30)^b^	45.24–3.09 (3.17–3.09)
Multiplicity	11.6(12.2)	25.6 (28.1)
Significance (<I>/σI)	30.1(3.6)	32.0 (5.5)
Total reflections	89,812(6867)	44,834 (3456)
No. unique reflections	7765(561)	1752 (123)
Completeness (%)	99.9(99.8)	100 (100)
*R_merge_* (%)^c^	4.8(74.6)	11.6 (80.4)
*R_p.i.m._* (%)^d^	1.5(22.9)	2.5 (15.5)
Anomalous completeness (%)		100 (100)
Anomalous multiplicity		15.5 (15.7)
Anomalous slope		1.643

*Refinement statistics*		
R_cryst_	20.8	
R_free_ (4.6% data)	23.7	

Nonhydrogen atoms		
All	637	
Water	212	

Mean overall B (Å^2^)	56.2	
RMSD from ideal values		
Bond distance (Å)	0.010	
Bond angle (degrees)	1.4	

a Diamond Light Source.

b Values in parentheses refer to the outer resolution shell.

c $R_{\mathrm{merge}}=\frac{\sum_{\mathrm{hkl}}\sum_{j}|I_{\mathrm{hkl}\text{,}j}-<I_{\mathrm{hkl}}>|}{\sum_{\mathrm{hkl}}\sum_{j}I_{\mathrm{hkl}\text{,}j}}$.

d $R_{p.i.m.}=\frac{\sum_{\mathrm{hkl}}\sqrt{\frac{1}{n-1}}\sum_{j=1}^{n}|I_{\mathrm{hkl}\text{,}j}-<I_{\mathrm{hkl}}>|}{\sum_{\mathrm{hkl}}\sum_{j}I_{\mathrm{hkl}\text{,}j}}$.

**Table 2:** Crystal structure of Tt1219^33-236^: data collection and refinement statistics.

	Native	Native	Iodide
*Data collection*			
Space group	*P*2_1_	*C*2	*C*2
Unit cell parameters	a = 65.4 Å, b = 66.9 Å, c = 97.5 Å; β = 99.1°	a = 116.8 Å, b = 32.1 Å, c = 108.1 Å; β = 115.5°	a = 116.3 Å, b = 32.0 Å, c = 108.4 Å; β = 115.4°
X-ray source and wavelength (Å)	DLS^a^ I24 (0.97872)	DLS IO4 (0.97)	DLS IO4 (1.2543)
Number of crystals	1	1	4
Resolution range (Å)	35.94–1.85 (1.90–1.85)^b^	57.99–2.30 (2.36–2.3)	57.77–2.27 (2.35–2.27)
Multiplicity	3.3 (3.4)	8.1 (8.0)	28.8 (9.2)
Significance (<I>/σI)	12.6 (2.2)	14.3 (2.6)	19.9 (2.7)
Total reflections	229,797 (17,727)	134,186 (9436)	491,706 (13,759)
No. unique reflections	70,620 (5223)	16,586 (1179)	17,086 (1490)
Completeness (%)	99.3 (99.8)	99.8 (99.2)	99.6 (96.2)
R_merge_ (%)^c^	7.6 (79.0)	10.9 (82.0)	80.2 (68.6)
R_p.i.m._ (%)^d^	6.4 (57.7)	4.4 (32.9)	13.8 (24.9)
Anomalous completeness (%)			99.6 (95.6)
Anomalous multiplicity			14.8 (4.8)
Anomalous slope			1.138

*Refinement statistics*			
R_cryst_	18.0	19.0	
R_free_ (4.6% data)	20.9	24.0	
Nonhydrogen atoms			
All	6264	3016	
Water	668	170	
Mean overall B (Å^2^)	27.6	45.0	
RMSD from ideal values			
Bond distance (Å)	0.018	0.021	
Bond angle (degrees)	1.8	1.9	

a Diamond Light Source.

b Values in parentheses refer to the outer resolution shell.

c $R_{\mathrm{merge}}=\frac{\sum_{\mathrm{hkl}}\sum_{j}|I_{\mathrm{hkl}\text{,}j}-<I_{\mathrm{hkl}}>|}{\sum_{\mathrm{hkl}}\sum_{j}I_{\mathrm{hkl}\text{,}j}}$.

d $R_{p.i.m.}=\frac{\sum_{\mathrm{hkl}}\sqrt{\frac{1}{n-1}}\sum_{j=1}^{n}|I_{\mathrm{hkl}\text{,}j}-<I_{\mathrm{hkl}}>|}{\sum_{\mathrm{hkl}}\sum_{j}I_{\mathrm{hkl}\text{,}j}}$.

**Table 3:** Crystal structure of Tt1222^38-123^: data collection and refinement statistics.

	Native	Iodide
*Data collection*		
Space group	*I*4_1_	*I*4_1_
Unit cell parameters	a = b = 84.4 Å, c = 97.2 Å	a = b = 84.3 Å, c = 97.1 Å
X-ray source and wavelength (Å)	DLS^a^ IO2 (0.9512)	DLS IO2 (1.5)
Number of crystals	1	1
Resolution range (Å)	31.86–1.85 (1.90–1.85)^b^	27.0–2.24 (2.30–2.24)
Multiplicity	6.9 (7.1)	9.1 (9.3)
Significance (<I>/σI)	34.2 (3.1)	12.6 (3.2)
Total reflections	197,443 (14,842)	149,665 (11,384)
No. unique reflections	28,582 (2087)	16,361 (1221)
Completeness (%)	99.0 (97.9)	99.9 (100)
*R_merge_* (%)^c^	2.7 (68.1)	10.5 (117.1)
*R_p.i.m._* (%)^d^	1.2 (29.5)	4.7 (41.4)
Anomalous completeness (%)		99.7 (100)
Anomalous multiplicity		4.5 (4.5)
Anomalous slope		1.198

*Refinement statistics*		
R_cryst_	18.3	
R_free_ (7.0% data)	20.8	

Nonhydrogen atoms		
All	1933	
Water	165	

Mean overall B (Å^2^)	44.8	
RMSD from ideal values		
Bond distance (Å)	0.012	
Bond angle (degrees)	1.5	

a Diamond Light Source.

b Values in parentheses refer to the outer resolution shell.

c $R_{\mathrm{merge}}=\frac{\sum_{\mathrm{hkl}}\sum_{j}|I_{\mathrm{hkl}\text{,}j}-<I_{\mathrm{hkl}}>|}{\sum_{\mathrm{hkl}}\sum_{j}I_{\mathrm{hkl}\text{,}j}}$.

d $R_{p.i.m.}=\frac{\sum_{\mathrm{hkl}}\sqrt{\frac{1}{n-1}}\sum_{j=1}^{n}|I_{\mathrm{hkl}\text{,}j}-<I_{\mathrm{hkl}}>|}{\sum_{\mathrm{hkl}}\sum_{j}I_{\mathrm{hkl}\text{,}j}}$.

**Table 4:** *T. thermophilus* pilin-like proteins: homologous structures.

Name	PDB	Organism	Function	r.m.s.d. (Å)^a^
***Tt1222**^**38-123**^*				
PilA4	4BHR	*T. thermophilus* HB8	Type IVa pilin	1.2
ComP	5HZ7	*Neisseria meningitidis*	Competence pilin	2.1
				
GspG	3G20	Enterohaemorrhagic *Escherichia coli*	T2SS	2.3
PilE	4NOA	*Pseudomonas aeruginosa*	Type IVa pilin	2.3
Pil_Bac1_	4D40	*Shewanella oneidensis*	Type IVa pilin	2.1
***Tt1218**^**36-123**^*				
EpsI	3CFI	*Vibrio vulnificus*	T2SS	2.1
GspI	3CI0	Enterotoxigenic *Escherichia coli*	T2SS	2.3

***Tt1219**^**33-236**^*				
XcpW	3NJE	*Pseudomonas aeruginosa*	T2SS	2.0
EpsJ	3CFI	*Vibrio vulnificus*	T2SS	2.7
GpsJ	3CI0	Enterotoxigenic *Escherichia coli*	T2SS	2.8

a Determined using the SSM alignment facility in CCP4MG (Potterton et al., 2004).

## Footnotes

1 *Abbreviations:* T4P, type IV pili; T2SS, type II secretion system; SEC, size exclusion chromatography.
