# Supplementary material for: Structures of type IV pilins from Thermus thermophilus demonstrate similarities with type II secretion system pseudopilins
Source: J Struct Biol. 2016 Dec;196(3):375–84. doi: 10.1016/j.jsb.2016.08.006 (PMC5131608; doi:10.1016/j.jsb.2016.08.006)
Supplement: Supplementary data 1 [file mmc1.pdf]

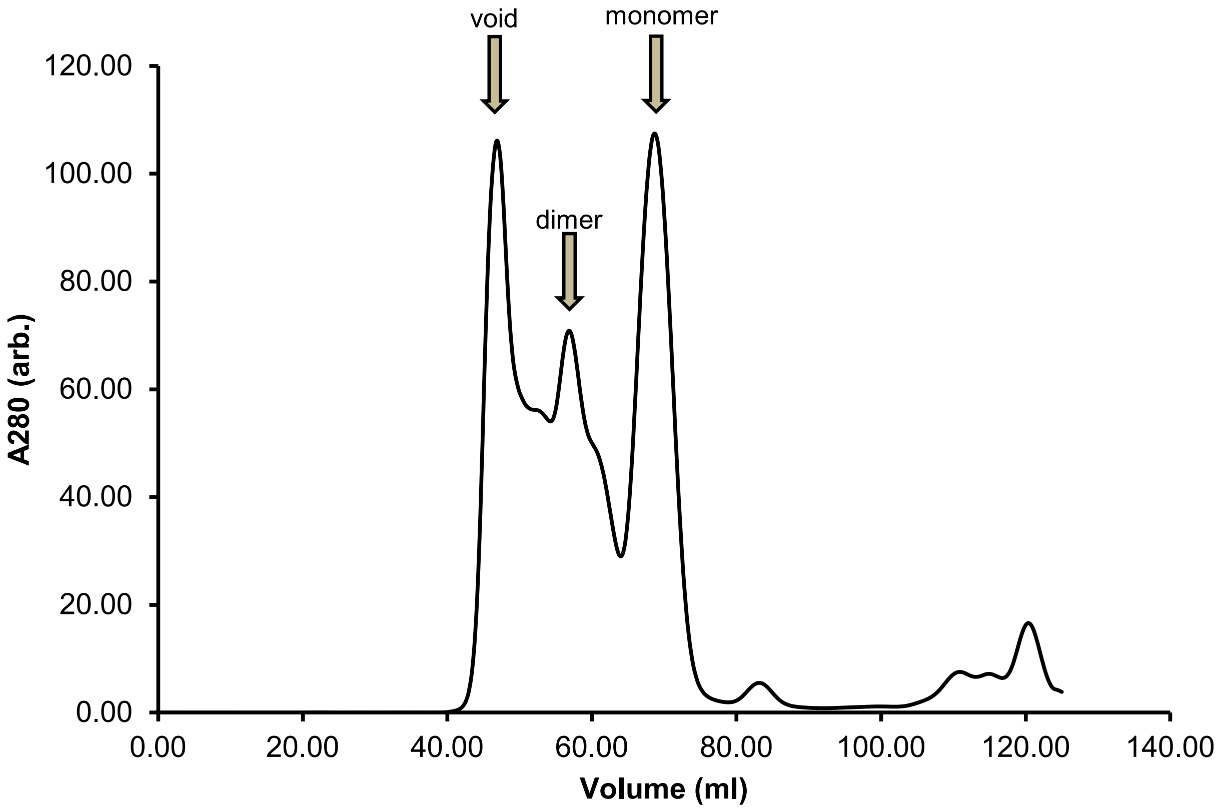

Supplementary Figure 1 Size exclusion chromatography elution profile for Tt1219$^{33\text{-}236}$; monomer, dimer and void elution volumes are indicated.
